# Cancer-associated fibroblasts are positively correlated with metastatic potential of human gastric cancers

**DOI:** 10.1186/1756-9966-29-66

**Published:** 2010-06-08

**Authors:** Kangkang Zhi, Xiaojun Shen, Hao Zhang, Jianwei Bi

**Affiliations:** 1 Department of General Surgery, the Second Military Medical University affiliated Changhai hospital, Shanghai 200433, China

## Abstract

**Background:**

The prognosis of gastric cancer patients is difficult to predict because of defects in establishing the surgical-pathological features. Cancer-associated fibroblasts (CAFs) have been found to play prominent role in promoting tumor growth, invasion and metastasis. Thus raises the hypothesis that the extent of CAFs prevalence may help to establish the prognosis of gastric cancer patients.

**Methods:**

Immunochemistry and realtime-PCR experiments were carried out to compare the expression of proteins which are specific markers of CAFs or secreted by CAFs in the tumor and normal tissue specimens. The extent of CAFs' prevalence was graded according to immunochemical staining, and correlation was further analyzed between CAFs' prevalence and other tumor characteristics which may influence the prognosis of gastric cancer patients.

**Results:**

Nearly 80 percent of normal gastric tissues were negative or weak positive for CAFs staining, while more than 60 percent of gastric cancer tissues were moderate or strong positive for CAFs staining. Realtime-PCR results also showed significant elevated expression of FAP, SDF-1 and TGF-β1 in gastric cancer tissues compared to normal gastric tissues. Further analysis showed that CAFs' prevalence was correlated with tumor size, depth of the tumor, lymph node metastasis, liver metastasis or peritoneum metastasis.

**Conclusions:**

Reactive cancer associated fibroblasts (CAFs) were frequently accumulated in gastric cancer tissues, and the prevalence of CAFs was correlated with tumor size, depth of the tumor and tumor metastasis, thus give some supports for establishing the prognosis of the gastric cancer patients.

## Background

Gastric cancer is the second leading cause of cancer-related death worldwide [1]. Substantial geographic variations exist in the incidence of gastric cancer and it represents the most common cancer in China [2]. More and more gastric cancer patients have been diagnosed in recent years with changing diet and lifestyle as well as developing diagnostic procedures. Although surgical treatment has shown to be effective for some early gastric cancers, including total gastrectomy and extended radical gastrectomy, the prognosis of these patients is poor due to the recurrence after surgery, in the form of lymphatic spread, blood-borne metastasis, or peritoneal dissemination [3].

The prognosis of patient with gastric cancer has been shown to be influenced by several established surgical-pathological features, such as the pathological stage, the location of the tumor and the histological type and grade of the tumor [4]. While Aurello et al. [5] have indicated that the number of nodes necessary to conclude N0 may vary according to the depth of tumor invasion (T), the TNM classification requires the retrieval and analysis of at least 15 lymph nodes for accurate staging. However, in most cases, the number of nodes dissected is smaller and only 20 to 30% of the patients have the recommended minimum dissection of 15 nodes. Accessorial indicators which can provide further information of the prognosis of gastric cancer patients are needed.

Cancer-associated fibroblast (CAF), one of the important stromal cells comprising solid tumors, has been found to play prominent role in promoting tumor growth and progression [6]. In contrast to resting fibroblasts, CAFs possess an activated phenotype and can be identified by their expression of fibroblast-specific protein 1 (FSP1), vimentin, desmin, and α-smooth-muscle actin [7]. CAFs communicate among themselves as well as with cancer cells and inflammatory and immune cells directly through cell contact and indirectly through paracrine/exocrine signaling, proteases, and modulation of the extracellular matrix (ECM). This complex communications network is pivotal to providing the appropriate microenvironment to support tumorigenesis, angiogenesis, and metastasis [8,9]. Additionally, compared to transformed tumor cells, CAFs are more genetically homogeneous [10] and it has been demonstrated by Gastavo et al that reactive stroma can act as a predictor of recurrence in prostate cancer [11], thus represent an attractive predictor and therapeutic target for tumor patients.

In this study, we collected 100 cases of surgical resection specimens of primary gastric cancer as well as normal gastric tissues (more than 5 cm far from tumor tissue) from January 2007 to June 2007 in the Second Military Medical University affiliated Changhai hospital (Shanghai, China). Immunochemistry and RT-PCR experiments were carried out to compare the expression of proteins which are specific markers of CAFs or secreted by CAFs in the tumor and normal tissue specimens. The extent of CAFs' prevalence was graded according to immunochemical staining, and correlation was further analyzed between CAFs' prevalence and other tumor characteristics which may influence the prognosis of gastric cancer patients.

## Methods

### Cohort Enrollment

One hundred cases of primary gastric cancer patients were enrolled from January 2007 to June 2007 in the Second Military Medical University affiliated Changhai hospital. All patients have provided a written informed consent. Entry criteria for this study include: (a) no preoperative chemotherapy treatment; (b) pathologically or cytologically validated gastric-adenocarcinoma; (c) aged between 18-85 years; (d) expected life>3 months; (e) WBC>3.5×10^9^/L; PLT>10^11^/L; Hb>100 g/L; Serum creatinine no more than 1.25 times of normal upper limit; GPT and ALP no more than 1.25 times of normal upper limit; Total bilirubin no more than 1.5 times of normal upper limit; PT<12s; and (f) no severe CNS disease.

### Pathological analysis

All specimens including tumor tissues and normal gastric tissues which was more than 5 cm far from tumor tissues were fixed in 10% formalin within 30 minutes after surgical resection. Paraffin embedded serial sections (4 μm) were prepared. Tumor differentiation was characterized according to WHO classification (2000) while the TNM classification was done according to International Union Against Cancer, fifth edition (1997).

### Immunochemistry

Antibody used in this procedure includes rabbit anti-FSP1 polyclonal antibody (Abcam, 1:50), mouse anti-α-SMA monoclonal antibody (Sigma, 1A4, 1:200), rat anti-procollagen I monoclonal antibody (Chemicon, Mab1912, 1:500), biotin-conjugated rat anti-mouse IgG polyclonal antibody (ebioscience, 13-4013, 1:100), biotin-conjugated mouse anti-rat IgG polyclonal antibody (ebioscience, 13-4813, 1:100) and biotin-conjugated mouse anti-rabbit IgG polyclonal antibody (BD PharMingen, C101-167, 1:100). Immunochemistry analysis was performed as previously described [12]. Briefly, paraffin sections were de-paraffinized in xylene and a series of graded alcohol solutions. The sections were then treated with 0.3% hydrogen peroxide (H2O2) in water for 10 minutes to quench any endogenous peroxidase activity within the tissue, and the nonspecific binding sites were blocked with 0.5% bovine serum albumin (BSA) for 10 minutes at room temperature. Next, the sections were incubated for 15 minutes in the presence of the primary antibody, and then the slides were washed in phosphate buffered saline (PBS) containing 0.1% Tween 20 (PBS/Tween) for 15 minutes while changing the solution 3 times before the application of the secondary biotinylated antibody. The slides were incubated with the secondary antibody for 15 minutes at room temperature before being washed for 15 minutes in PBS/Tween that was changed 3 times. The sections were then incubated for 15 minutes with an avidin-biotinylated horseradish peroxidase complex, and the reaction visualized using 0.02% 3,3'-diaminobenzidine tetrahydrochloride as a chromogen in a Tris-HCl buffer, pH 7.6, containing 0.03% H2O2. Hematoxylin was used to counterstain the nuclei.

### Histological analysis

To evaluate the level of FSP1, α-SMA and procollagen-I expression, the percentage of positive-staining cells were graded on a scale of 0-3, with less than 5% positive-staining cells as grade 0, 5-25% as grade 1, 26-50% as grade 2, and more than 50% as grade 3. And the intensity of staining also graded on a scale of 0-2, with negative to weak intensity as grade 0, weak to moderate intensity as grade 1, and moderate to strong intensity as grade 2. Ten high-power fields were selected randomly for each slides and analyzed by two pathologists independently. For each marker, the score of percentage and intensity was multiplied and the scores for these three markers was added when these markers was analyzed conjointly. And the final score between 0-6 was determined as negative (-), score between 7-9 was determined as weak positive (+), score between 10-12 was determined as moderate positive (++), and score higher than 13 was determined as strong positive (+++).

### Realtime-PCR

Total RNA was extracted from tumor or normal tissues by Trizol reagent (invitrogen) and first-strand cDNA was synthesized using RevertAid First Strand cDNA Synthesis Kit (Fermentas, USA) as described previously [13]. Realtime PCR was carried out using LightCycler DNA Master SYBR Green I Kit (Roche Diagnostics, Mannheim, Germany) according to the manufacturer's instructions. The copies of target cDNA were normalized by GAPDH expression. Primers for FAP, SDF-1, TGF-β1 and GAPDH were listed as follows:

FAP F: 5'-TGGGAATATTACGCGTCTGTCTAC-3'

FAP R: 5'-GATAAGCCGTGGTTCTGGTCA-3'

SDF-1 F: 5'-CCGTCAGCCTGAGCTACA-3'

SDF-1 R: 5'-GAAGGGCACAGTTTGGAG-3'

TGF-β1 F: 5'-GCAACAATTCCTGGCGATAC-3'

TGF-β1 R: 5'-AAGGCGAAAGCCCTCAAT-3'

GAPDH F: 5'-ATCAAGTTGCGTGCTGTG-3'

GAPDH R: 5'-TGCGAAATGAAAGGAGTGT-3'

For each target cDNA, the copies of normal tissue samples is averaged, and the copies of each tumor tissue sample is divided by the average, then the results of these three target cDNA is added for each tumor tissue sample. If the sum is equal to or larger than 8, then the tumor tissue is considered to be positive for CAFs.

### Statistical analysis

Data are shown as means and standard deviations. Statistical analyses of the data were analyzed with the two-tailed independent Student's t test and χ^2 ^analysis by SPSS 12.0. The level of statistical significance was set at P < 0.05.

## Results

### Reactive tumor associated fibroblasts were prevalent in gastric cancer tissues

To determine the extent of CAFs' prevalence in gastric cancer tissues, paraffin embedded sections of tissue specimens were prepared and stained for FSP1, α-SMA and procollagen I expression as described above. In addition, realtime-PCR was carried out to determine the expression level of several proteins which was expressed or secreted by reactive CAFs, such as FAP, SDF-1 and TGF-β1.

**Figure 1:**
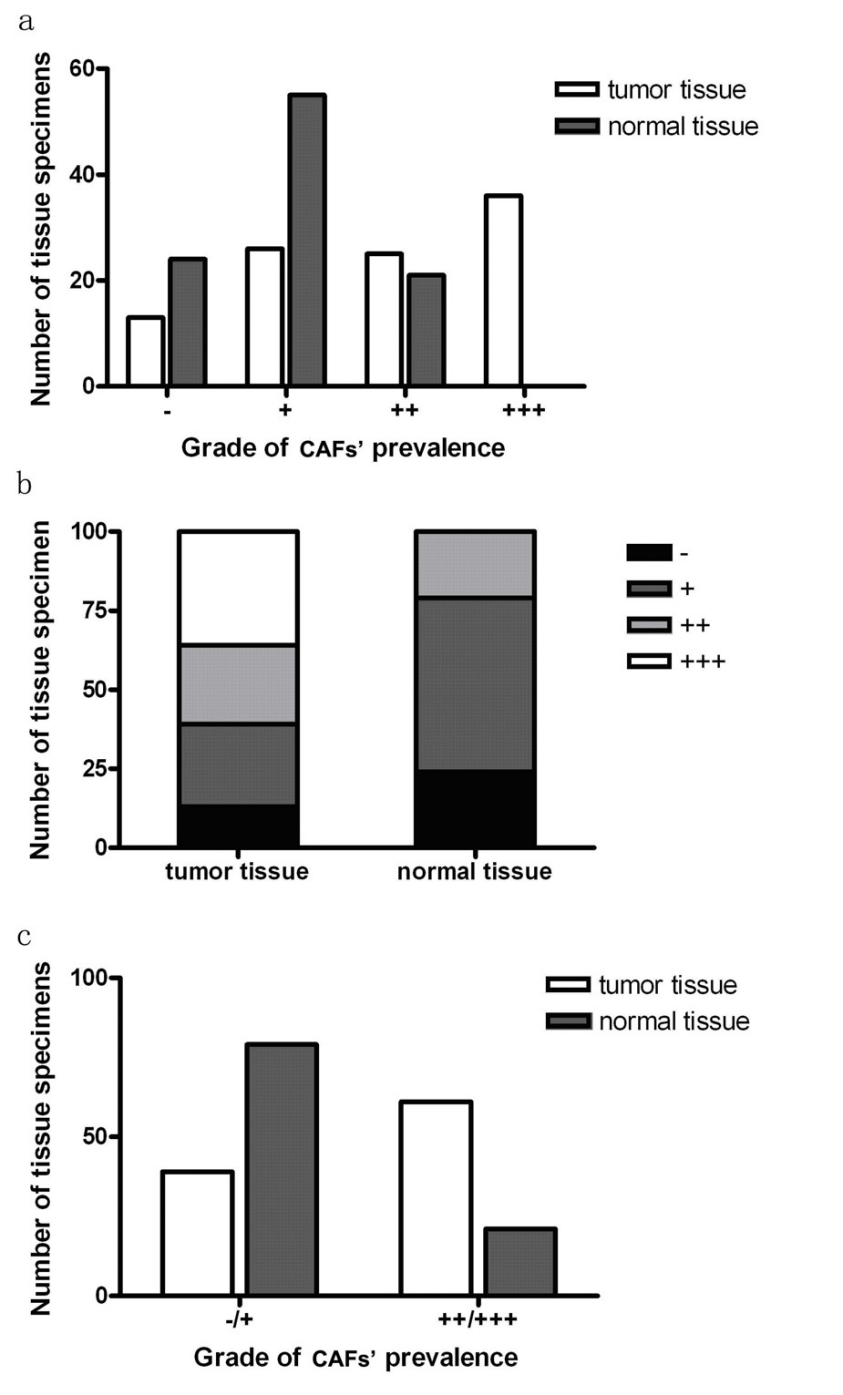
**Immunochemistry analysis of the grade of CAFs' prevalence in tumor and normal gastric tissues**. Paraffin sections of surgically resected tumor and normal tissues from the same gastric cancer patients (100 cases) were stained for FSP1, α-SMA and procollagen-1 expression and CAFs prevalence was graded according to the positive rate and intensity of the immunochemical staining. The number of tumor or normal tissue specimens graded as -, +, ++ and +++ was compared (a). And the distribution of these four grades of CAFs' prevalence in the 100 tumor or normal tissue specimens were analyzed (b). Grade - and + was regarded as negative, while grade ++ and +++ was regarded as positive for CAFs prevalence, then the number of the tumor or normal tissue specimens which was positive or negative for CAFs' prevalence was compared (c).

Results of immunochemistry staining showed that more reactive fibroblasts were present in gastric cancer tissues than normal gastric tissues. Twenty four out of the 100 normal specimens were negative (-) for reactive fibroblasts staining and 55 normal specimens were weak positive (+). And the number of normal specimens which were moderate (++) or strong positive (+++) were 21 and 0, respectively. While concerning cancer tissues, there were 13, 26, 25 and 36 specimens which were negative (-), weak positive (+), moderate positive (++) and strong positive (+++) for fibroblast staining, respectively (Fig 1a and Fig 1b). And if tumor specimens graded as negative or weak positive were regarded as negative, and moderate or strong positive were regarded as positive, there was a significant difference between tumor and normal tissues concerning the positive rate of CAFs (Fig 1c).

**Figure 2:**
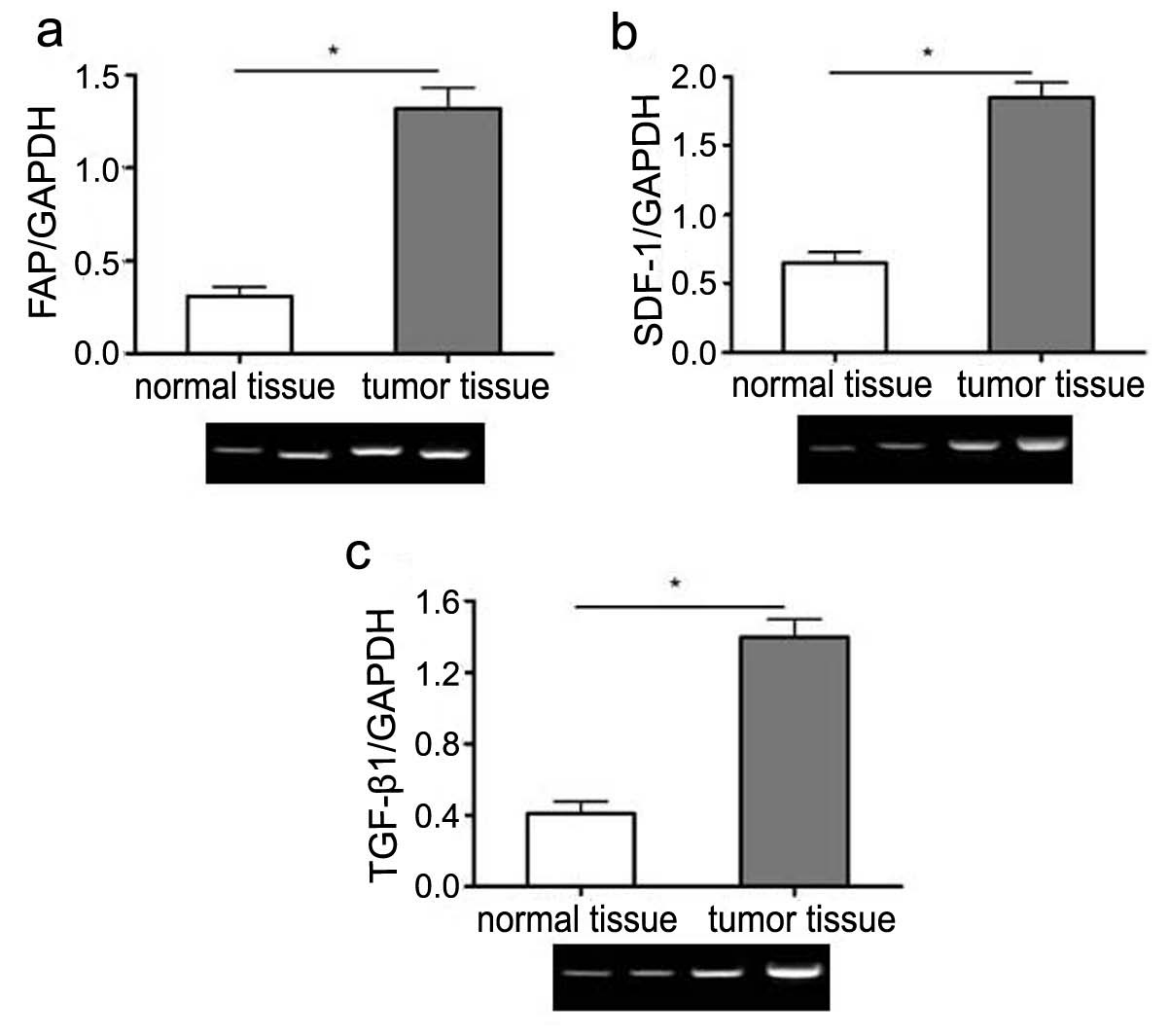
**Realtime-PCR analysis of secreted proteins by CAFs in tumor and normal gastric tissues**. Total RNA was extract and cDNA was prepared from surgically resected tumor and normal tissues from the same gastric cancer patients (100 cases). Realtime-PCR was carried out to compare the expression level of FAP (a), SDF-1 (b) and TGF-β1 (c) in tumor and normal tissues, the first two lanes of the electrophoretogram represented normal tissues and the last two lanes represented tumor tissues. *:p < 0.01.

For mRNA expression of the proteins, results showed that the expression level of all these proteins were elevated in tumor specimens compared to these in normal tissues. Taking FAP as an example, the mRNA expression level of FAP in tumor specimens was 4 times higher than that in normal tissues (Fig 2a). And there were also 3 times elevation of mRNA expression level regarding SDF-1 (Fig 2b) or TGF-β1 (Fig 2c).

From these results, we can conclude that reactive CAFs were prevalent in gastric tumor tissues and secret high level of proteins which have been demonstrated to be essential for tumor growth, invasion and metastasis.

### CAFs' prevalence was closely related with invasive and metastatic properties of gastric tumor

To determine whether the grade of CAFs' prevalence can be served as a predictor for the prognosis of gastric cancer patients, correlation analysis was carried out between the grade of CAFs' prevalence and other clinicopathological parameters of gastric cancers. Tumor specimens graded as negative or weak positive were regarded as negative, and moderate or strong positive were regarded as positive in these analysis. Patient and tumor characteristics were described in Table 1. We can also find in Table 1 that there was no correlation between CAFs' prevalence and age, gender of the patient or the location of the tumor. There was an increase of CAFs' prevalence when the tumor differentiation decreased from well-differentiated (43.75%) to poorly-differentiated (64.00%), while the positive rate of CAFs in undifferentiated gastric cancer is only 26.67%, much less than that in well or poorly differentiated gastric cancers, thus we could not find the correlation between the CAFs' prevalence and tumor differentiation (P = 0.56). While concerning tumor size, depth of the tumor (T) and lymph node metastasis (N), there showed statistically significant correlation between the prevalence of CAFs and these tumor characteristics, with higher positive rate of CAFs in larger tumors, more invasive tumors and tumors with more lymph node metastasis. Also we can find that the positive rate of CAFs was high in gastric cancers with liver metastasis (P < 0.01) or peritoneum metastasis (P < 0.01).

**Table 1:** Patient and tumor characteristics and their relationship with CAFs prevalence

	N	Positive for CAFs N (%)	P value
Age (year)			2.77^a^
≤60	47	22 (46.81)	
>60	53	29 (54.72)	
Sex			5.11^a^
Male	57	32 (56.14)	
Female	43	19 (44.19)	
Location of the tumor			1.35^b^
Proximal end of stomach (1/3)	13	9 (69.23)	
Gastric body (1/3)	19	9 (47.37)	
Remote end of stomach (1/3)	51	22 (43.14)	
More than 1/3 of the stomach involved	17	11 (64.71)	
Tumor differentiation			0.56^b^
Well differentiated	16	7 (43.75)	
Moderate differentiated	44	24 (54.55)	
Poorly differentiated	25	16 (64.00)	
Undifferentiated	15	4 (26.67)	
Tumor size			0.02^a^
≤5 cm	62	16 (35.48)	
>5 cm	38	29 (76.32)	
Depth of tumor (T)			0.03^b^
Tis	4	1 (25.00)	
T1	13	5 (38.46)	
T2	39	19 (48.72)	
T3	26	15 (57.69)	
T4	18	11 (61.11)	
Lymph node metastasis (N)			<0.01^a^
N0	46	16 (34.78)	
N1-3	54	35 (64.81)	
Liver metastasis			<0.01^a^
Yes	12	9	
No	88	42	
Peritoneum metastasis			<0.01^a^
Yes	9	7 (77.77)	
No	91	44 (48.35)	
TNM Stage			<0.01^b^
IA	15	3 (20)	
IB	7	2 (28.57)	
II	19	6 (31.58)	
IIIA	23	11 (47.83)	
IIIB	15	8 (53.33)	
IV	21	14 (66.67)	

a: Fisher exact test; b: Chi-Square Tests

**Figure 3:**
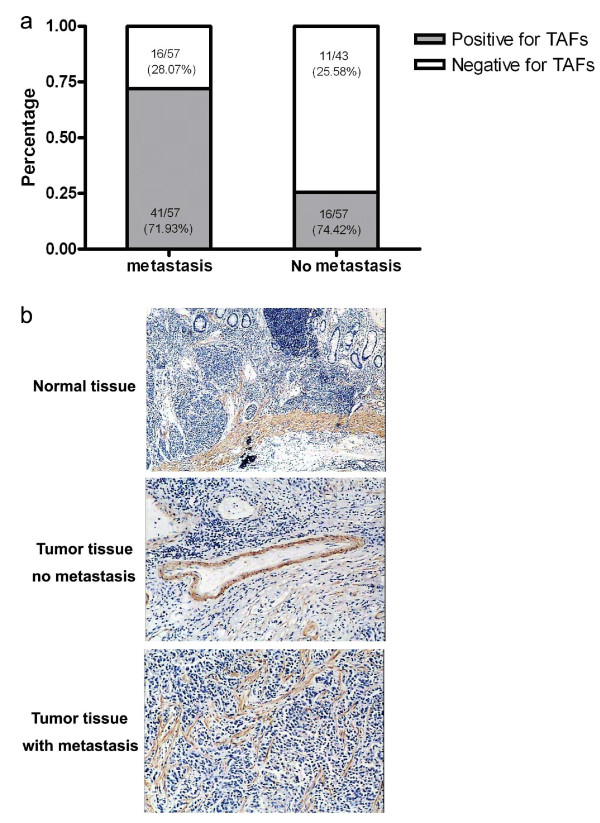
**The percentage of tumor specimens which was positive or negative for CAFs' prevalence in the group of patients with or without tumor metastasis**. The tumor specimens were grouped according to whether or not the gastric cancer patients had tumor metastasis (whatever lymph node metastasis, distant metastasis or organ metastasis). And the percentage of the specimens which was positive (grade - or + according to immunochemical staining) or negative (grade ++ or +++ according to immunochemical staining) for CAFs' prevalence was analyzed (a). And the immunochemical staining of α-SMA was shown in normal gastric tissue, gastric cancer tissue without metastasis and gastric cancer tissue with metastasis (b).

In addition, in the situation of tumor metastasis, whatever lymph node metastasis, distant metastasis or organ metastasis, the positive percentage for CAFs is much higher than that in those without metastasis (71.93% vs 25.58%, P < 0.01) (Fig 3).

And we also analyzed the correlation between the mRNA level of FAP, SDF-1 and TGF-β1 and the gastric cancer stage. The level of these proteins were scored as described in the methods and the tumor tissue samples were determined to be positive if the score is equal to or larger than 8. It was found that the positive percentage is much high in large tumors (>5 cm, 32/38) than that in small tumors (≤5 cm, 20/62) (p < 0.05). And the positive percentage in tumor samples with TNM stage IA, IB, II, IIIA, IIIB and IV are 33.3% (5/15), 42.9%(3/7), 52.6%(10/19), 60.9%(14/23), 73.3%(11/15) and 76.2(16/21), respectively, showing that the prevalence of CAFs is closely correlated with the gastric cancer stages (p < 0.01). These results strongly suggested that CAFs' prevalence could help to establish the gastric cancer stage and could be used as a marker for the prognosis of gastric cancer patients.

## Discussion

Recent studies in molecular and cellular biology have shown that tumor growth and metastasis are not determined by cancer cells alone but also by a variety of stromal cells [14,15]. The stroma actively provides continuous support to carcinoma cells throughout the different pathophysiological processes that modulate tumor progression. Fibroblasts are an important component of tumor stroma, which have received increased attention because of their participation in tumor development, including growth, invasion and metastasis, such as in prostate cancer [16,17] or breast cancer [18,19]. It has also been demonstrated in a gastric cancer mice model that activated fibroblasts promote tumor angiogenesis [20], and it is consistent with out results that activated fibroblasts were accumulated in human gastric cancer tissues.

The term fibroblast encompasses a number of stromal cells with a broadly similar phenotype. Most tumors incorporate an obvious biologically active, fibroblastic cell type known variously as reactive fibroblasts, myofibroblasts, or simply tumor-associated fibroblasts. Smooth muscle α-actin (α-SMA) is the most common marker used to identify CAFs, while its expression can also be found in smooth muscle cells and myoepithelial cells [21]. So other markers should be used in combination with α-SMA to identify CAFs. Fibroblast-specific protein 1 (FSP1, S100A4), a member of the family of Ca2+ -binding S100 proteins, constitutively expressed in the cytoplasm of tissue fibroblasts, and its expression is highly specific for fibroblasts [22,23]. It is widely accepted to combine a-SMA and FSP1 for the identification of tumor-associated fibroblasts. And in our experiment, we also used a third marker, procollagen I, to identify reactive CAFs with production of extracellular matrix components.

We also detected the mRNA expression level of other proteins which is expressed or secreted by CAFs. FAP is a type II transmembrane cell surface protein belonging to the post-proline dipeptidyl aminopeptidase family, with dipeptidyl peptidase and endopeptidase activity, including a collagenolytic activity capable of degrading gelatin and type I collagen [24,25]. FAP is expressed selectively by CAFs and pericytes in more than 90% of human epithelial cancers examined [26-30] and research has been reported in animal model showing a therapeutic effect by inhibiting FAP expression or enzymatic activity [31]. The next protein we selected to detect is SDF-1, which is secreted by CAFs and stimulates tumor cells proliferation, angiogenesis, invasion and metastasis through the CXCR4 receptor expressed by tumor cells [32-34]. Another secreted protein we detected is TGF-β1, which is a potent inducer for myofibroblasts differentiation [35], and may play a role in tumor invasion-metastasis cascades [36]. The results of the present study showed that these proteins were up-regulated in gastric cancer tissues, suggesting their potential role in promoting gastric cancer progression.

Gastric cancer is the second leading cause of cancer-associated mortality in the world. Prognosis in patients with gastric cancer is difficult to establish because it is commonly diagnosed when gastric wall invasion and metastasis have occurred. Several groups attempted to find some biomarkers for the prognosis of gastric cancer. For example, the expression of several extracellular matrix metalloproteinases (MMP-2, 7, 9) has been found to be elevated in gastric cancer tissues compared to healthy gastric tissues. And the up-regulation of these MMPs in gastric cancer has been associated with a poor prognosis and elevated invasive capacity [37]. Another example is insulin-like growth factor-1 receptor (IGF-1R), it was frequently expressed in gastric cancers and was associated with tumor size, quantity of stroma, depth of wall invasion, lymph node metastasis, TNM stages and differentiation status of gastric cancer [38]. And VEGF-C expression at tumor margins was also associated with nodal metastasis, lymphatic vessel invasion, poor recurrence-free survival, and poor overall survival, and could serve as an independent predictor for patients with gastric carcinoma [39]. We can find that these predictors are either proteins secreted by CAFs or receptors expressed by tumor cells which bind the proteins secreted by CAFs, so these clues may suggest that the prevalence of CAFs in gastric cancer tissues will be an important predictor of gastric cancer patients. And our results confirmed that the prevalence of CAFs was closely associated with the metastatic potential of gastric cancer, and further work should be done to confirm the correlation between CAFs' prevalence and survival of gastric cancer patients.

## Conclusions

Our findings report here demonstrate that reactive cancer associated fibroblasts (CAFs) were frequently accumulated in gastric cancer tissues, and the prevalence of CAFs was correlated with tumor size, depth of the tumor and tumor metastasis as well as the overall TNM stage, suggesting that CAFs were critical for tumor growth, invasion and metastasis, thus give some supports for the prognosis of the gastric cancer patients.

## Abbreviations

CAFs: tumor associated fibroblasts; FAP: fibroblast activation protein; SDF-1: stromal-cell derived factor 1; TGF-β1: transforming growth factor beta 1; ECM: extracellular matrix; WBC: white blood cell count; PLT: platelet count; Hb: hemoglobin; GPT: glutamic-pyruvic transaminase; ALP: alkaline phosphatase; PT: prothrombin time; CNS: central nerves system; α-SMA: α-smooth-muscle actin; FSP1: fibroblast specific protein 1; GAPDH: glyceraldehyde phosphate dehydrogenase; MMP: matrix metalloproteinase; VEGF: vascular endothelial growth factor.

## Competing interests

The authors declare that they have no competing interests.

## Authors' contributions

KK Zhi carried out the specimen collection and immunochemistry experiment. XJ Shen dealed with RNA extraction and realtime PCR. H Zhang carried out the statistical analysis. JW Bi designed the study and helped to draft the manuscript. All authors have read and approved the final manuscript.
